# Retraction Note: Molecular detection of pathogenic *Escherichia coli* strains and their antibiogram associated with risk factors from diarrheic calves in Jimma Ethiopia

**DOI:** 10.1038/s41598-021-98353-6

**Published:** 2021-09-14

**Authors:** Destaw Asfaw Ali, Tesfaye Sisay Tesema, Yosef Deneke Belachew

**Affiliations:** 1 College of Veterinary Medicine and Animal Sciences, University of Gondar, Gondar, Ethiopia; 2 Institute of Biotechnology, Addis Ababa University, Addis Ababa, Ethiopia; 3 College of Agriculture and Veterinary Medicine, Jimma University, Jimma, Ethiopia

Retraction of: *Scientific Reports* 10.1038/s41598-021-93688-6, published online 13 July 2021

The Editors have retracted this Article.

After publication of this Article, concerns were brought to the attention of Editors regarding some of the data presented. The Editors requested the original data for this study. However, the data provided by the Authors for Figure 1 and Tables 3 and 4 could not be unambiguously ascribed to the experiments described. As these data are the basis for the main conclusions of the study, the Editors no longer have confidence that the conclusions presented are supported.

Destaw Asfaw Ali, Tesfaye Sisay Tesema and Yosef Deneke Belachew disagree with this retraction.

